# Gastrointestinal Bleeding Due to NOACs Use: Exploring the Molecular Mechanisms

**DOI:** 10.3390/ijms232213955

**Published:** 2022-11-12

**Authors:** Angela Saviano, Mattia Brigida, Carmine Petruzziello, Marcello Candelli, Maurizio Gabrielli, Veronica Ojetti

**Affiliations:** 1 Department of Emergency Medicine, Fondazione Policlinico Universitario A. Gemelli, 00168 Rome, Italy; 2 Gastroenterology Unit, Department of Systems Medicine, Tor Vergata University, 00133 Rome, Italy; 3 Department of Emergency Medicine, San Carlo di Nancy Hospital, GVM Research, 00165 Rome, Italy; 4 Department of Emergency Medicine, Catholic University of the Sacred Heart, 00168 Rome, Italy

**Keywords:** novel oral anticoagulants, NOACs, gastrointestinal bleeding, edoxaban, apixaban, rivaroxaban, dabigatran, warfarin

## Abstract

Novel oral anticoagulants (NOACs) are drugs approved for the prevention and treatment of many thromboembolic cardiovascular conditions as a safer alternative to warfarin. We reviewed studies published in PubMed^®^, UpToDate^®^, Web of Science^®^, and Cochrane^®^ about NOACs’ risks and benefits in patients requiring anticoagulation, with a focus on gastrointestinal bleeding and on molecular and pathophysiological mechanisms underlying the risk of bleeding in patients treated with them. Apixaban resulted in a lower rate of gastrointestinal bleeding compared to dabigatran and rivaroxaban. However, data reported that gastrointestinal bleeding in patients treated with NOACs was less severe compared to warfarin. Studies show promising results on the increased and widespread use of NOACs in patients who require anticoagulation (for example—in case of atrial fibrillation or high risk of venous thromboembolism), reporting an overall lower risk of major bleeding events. The profile of NOACs was more effective and secure compared to warfarin, but a more careful medical prescription is required in patients who are at high risk of gastrointestinal bleeding.

## 1. Introduction

### 1.1. Novel Oral Anticoagulants: Molecular Structure and Targets, Indications and Side Effects

Novel oral anticoagulants (NOACs) are drugs selective for thrombin or activated factor Xa approved for the prevention and treatment of many thromboembolic conditions [1]. They included venous thromboembolism in patients undergoing orthopedic surgery, such as hip or knee arthroplasty, atrial fibrillation, stroke prevention in nonvalvular patients, and pulmonary embolism. NOACs, such as apixaban, edoxaban, dabigatran, rivaroxaban and betrixaban, have become an alternative to vitamin K anticoagulants (i.e., warfarin). Nowadays, they are largely used in many countries. 

Despite all four drugs being termed NOACs, dabigatran has a unique mechanism of action targeting thrombin, whilst rivaroxaban, edoxaban, and apixaban all target factor Xa [2]. However, from the chemical point of view, each has its unique molecular structure. Dabigatran is an aromatic amide, a member of benzimidazoles, a carboxamidine, a member of the pyridines, and a beta-alanine derivative. Its molecular weight is 471.5 Da, and it is obtained by formal condensation of the carboxy group of 2-{[(4-carbamimidoylphenyl)amino]methyl}-1-methyl-1H-benzimidazole-5-carboxylic acid with the secondary amino group of N-pyridin-2-yl-beta-alanine [2,3]. Rivaroxaban is a member of thiophenes, an organochlorine compound, an oxazolidinone, a member of morpholines, a lactam, an aromatic amide, and a monocarboxylic acid amide. Its molecular weight is 435.9 Da, and it is obtained by formal condensation of the carboxy group of 5-chlorothiophene-2-carboxylic acid with the amino group of 4-{4-[(5S)-5-(aminomethyl)-2-oxo-1,3-oxazolidin-3-yl]phenyl}morpholin-3-one [2,4]. Edoxaban is a monocarboxylic acid amide, a chloropyridine, a thiazolopyridine, and a tertiary amino compound that is N’-(5-chloropyridin-2-yl)-N-[(1S,2R,4S)-4-(dimethylcarbamoyl)-2-[(5-methyl-6,7-dihydro-4H-[1,3]thiazolo [5,4-c]pyridine-2-carbonyl)amino]cyclohexyl]oxamide, with a molecular weight of 548.1 Da [2,5]. Apixaban is a pyrazolopyridine, a member of piperidones, a lactam, and an aromatic ether, that is 7-oxo-4,5,6,7-tetrahydro-1H-pyrazolo[3,4-c]pyridine-3-carboxamide substituted at position 1 by a 4-methoxyphenyl group and at position 6 by a 4-(2-oxopiperidin-1-yl)phenyl group, with a molecular weight of 459.5 Da [2,6].

These drugs emerged as more safe, convenient, and effective therapies for the prevention and treatment of patients with some cardiovascular thromboembolic diseases [7] compared to warfarin. It is well known that an appropriate use of these novel anticoagulants requires knowledge of patients’ characteristics, age, renal and liver function, state of pregnancy, concomitant medications (most of NOACs are substrates of P-glycoprotein with a potential risk of drug–drug interactions) [8], and benefits and risks. In particular, patients taking NOACs have a higher risk of bleeding (intracranial hemorrhage, gastrointestinal bleeding, genitourinary, and respiratory) compared to people that are not on anticoagulants [9]. Although, as concerns gastrointestinal bleeding, many studies reported that the risk of gastrointestinal bleeding due to NOACs use was less frequent than warfarin. The largest reduction in upper gastrointestinal bleeding was observed in patients taking apixaban and edoxaban (compared to vitamin K antagonists) [1,7,10]. Warfarin, as a vitamin K antagonist (VKAs), acts indirectly by inactivating the clotting factors II, VII, IX, and X (and natural anticoagulant proteins C and S), while NOACs, as described above, target the specific factors IIa and Xa [11], acting both on the intrinsic and extrinsic coagulation pathways.

### 1.2. Literature Studies about NOACs and Gastrointestinal Bleeding

A study performed by Lee et al. [12] reported that, among the four NOACs, the difference in upper gastrointestinal bleeding risk was not statistically significant. On the contrary, another study by Yang et al. [13] revealed that rivaroxaban and dabigatran resulted in more gastrointestinal bleeding compared to apixaban. The prevalence of gastrointestinal bleeding in patients treated with warfarin was 2.5–10.1% [14]. A meta-analysis performed by Loffredo et al. [15] suggested that patients at high risk of gastrointestinal bleeding should avoid rivaroxaban and high doses of dabigatran and edoxaban. The authors selected four studies including 71,302 patients. They found that NOACs increased gastrointestinal bleeding in patients with high risk factors. Rivaroxaban and high doses of edoxaban and dabigatran significantly increased the risk of gastrointestinal bleeding compared to warfarin, while no effects were detected with apixaban [15]. A study by Gu et al. [16] revealed that NOAC use was associated with a risk of gastrointestinal bleeding comparable or higher than that of warfarin. An Asian study (more than 200,000 patients) by Yang et al. [17] found that NOACs were safer in patients with atrial fibrillation compared to warfarin as regards gastrointestinal bleeding. The topic of NOACs related to gastrointestinal bleeding has been discussed for years because multiple adjustments for sex, age, baseline comorbidities, and concomitant medications are needed to perform a correct statistical evaluation of overall risks. Another study [12] revealed that the association between NOACs and proton pump inhibitors (PPI) resulted in a lower upper gastrointestinal bleeding risk compared to warfarin. In particular, a significant reduction of gastrointestinal bleeding was observed in high-risk patients taking edoxaban and apixaban compared to warfarin [12]. A study by Radaelli et al. [18] found that the risk of gastrointestinal bleeding in patients with NOACs was uncertain, and the reported incidence of gastrointestinal bleeding was heterogeneous. Xu et al. [1] suggested a higher risk of recurrence in bleeding with NOAC assumption and resumption. In particular, their study reported a 50% increase in the risk of gastrointestinal bleeding in patients assuming dabigatran compared to warfarin, or a more than twofold higher bleeding-risk with rivaroxaban compared to warfarin [19]. Larsen et al. [20] assessed the efficacy and safety of dabigatran compared to warfarin for atrial fibrillation, finding that the incidences of both major and gastrointestinal bleeding were similar.

### 1.3. Literature Studies about NOACs and Major Bleeding

Lin et al. [21] reported that dabigatran was associated with lower major bleeding risk than warfarin in patients with atrial fibrillation, as well. A study by Yao et al. [22] revealed that apixaban had a lower risk of major bleeding—both intracranial and gastrointestinal—compared with warfarin. On the contrary, no significant difference in the risk of gastrointestinal bleeding was found between dabigatran and warfarin. As regards rivaroxaban, it was associated with a similar risk of major bleeding and a higher risk of gastrointestinal bleeding compared to warfarin [22]. Finally, regular-dose apixaban had a lower risk of major bleeding compared to warfarin, whereas reduced-dose apixaban had a similar risk of major bleeding [22]. Another Korean study made a comparison of the safety and effectiveness between edoxaban and rivaroxaban, showing that edoxaban resulted in less gastrointestinal bleeding [23]. The use of proton pump inhibitors can protect against major bleeding in NOAC patients [7]. Most of cases required NOAC dose adjustment or a temporary discontinuation of the drugs [14]. Only a few cases of fatal bleeding with hypovolemic shock or dangerous organ ischemia have been reported [14]. Miller et al. [24] performed a meta-analysis that included 28 randomized controlled trials (RCT) with a total of 129,357 patients. They compared major gastrointestinal bleeding of NOACs (all pooled together) vs. standard treatments (warfarin, enoxaparin, or aspirin), showing a rate of gastrointestinal bleeding of 1.5%/year vs. 1.3%/year. A more recent meta-analysis analyzed 43 RCTs, with a total of 183,752 patients, and it also found a similar rate of major gastrointestinal bleeding for NOACs (1.19%/year vs. 0.92%/year) compared to warfarin. There were also no significant differences for upper gastrointestinal bleeding (0.85% for NOACs vs. 0.61% for warfarin) and lower gastrointestinal bleeding (0.58% vs. 0.50%, respectively) [16]. More specifically, as regards dabigatran, the RE-LY trial [25] showed that only high doses of dabigatran (150 mg twice a day) was associated with a higher risk of major gastrointestinal bleeding when compared to warfarin (1.51%/year vs. 1.02%/year). With a dose of dabigatran of 110 mg twice a day, the risk of major gastrointestinal bleeding decreased to 1.12%. However, when conducting an age-related analysis for people aged <75 years, the risk of gastrointestinal bleeding was similar to warfarin for both dosage of dabigatran (1.22% for 150 mg and 0.84% for 110 mg), confirming that 150 mg twice a day is the appropriate dose due to a lower risk of stroke and a similar risk of GI bleeding. As regards edoxaban, the ENGAGE AF-TIMI 48 trial [26] showed that the risk of major gastrointestinal bleeding was increased in the higher dose of edoxaban (60 mg) compared to warfarin (1.51%/year vs. 1.23%/year). Meanwhile, it was significantly decreased in the lower dose (30 mg, 0.82% year). Concerning rivaroxaban, the ROCKET AF trial [27] showed that rivaroxaban has a higher risk of gastrointestinal bleeding compared to warfarin (2.0% vs. 1.24%/year). Another trial conducted on 5868 patients showed that rivaroxaban was associated with a higher overall risk of gastrointestinal bleeding compared to apixaban and dabigatran in all patients. Finally, for apixaban, the ARISTOTLE trial [28] showed a similar rate of major gastrointestinal bleeding between apixaban and warfarin (0.76 % vs. 0.86%/year), with a similar rate of upper gastrointestinal bleeding (0.43% vs. 0.56%) and lower gastrointestinal bleeding (0.25% vs. 0.24%) compared to warfarin.

### 1.4. Aims and Methods

We reviewed studies published in PubMed^®^, UpToDate^®^, Web of Science^®^, and Cochrane^®^ about NOACs’ risks and benefits in patients requiring anticoagulation, with the aim of focusing on gastrointestinal bleeding and on molecular and pathophysiological mechanisms underlying the risk of bleeding in patients treated with them.

## 2. NOACs and GI Bleeding: Molecular and Pathophysiological Aspects

### 2.1. Pathophysiology of Gastrointestinal Bleeding under NOACs Treatment

**Figure 1:**
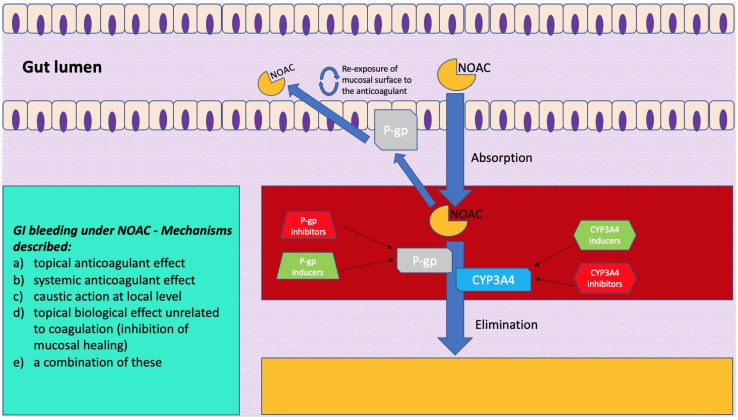
A simplification model of how molecular mechanisms related to pharmacokinetics of novel oral anticoagulants (NOACs) exert a clinical impact on gastrointestinal bleeding. On the left is a scheme showing mechanisms described for gastrointestinal bleeding under NOAC treatment.

The digestive tract is endowed with a rich intra and submucosal vascularization. However, even in conditions of wellbeing, the gastrointestinal mucosa is regularly damaged. In this regard, endoscopic data on healthy volunteers allowed us to note how gastric and small bowel erosions are present in 5–10% and 10–15% of cases, respectively. This mucosal vulnerability, which is the expression of the impact of acid and digestive enzymes such as amylase, trypsin and pepsin, as well as exogenous bacterial flora, makes intestinal vascularization prone to clinical or sub-clinical bleeding. This bleeding tendency is, however, modified by the intake and the consequent effect of anticoagulants, and various mechanisms have been described (Figure 1): (a) topical anticoagulant effect, (b) systemic anticoagulant effect, (c) caustic action at local level, (d) topical biological effect not correlated to coagulation (inhibition of mucosal healing), or (e) a combination of these [29,30,31]. While, for warfarin, which is 95% absorbed, only a systemic effect has been described, and the unabsorbed fraction does not appear to cause topical damage. For NOACs, more mucosal damage mechanisms have been highlighted. First of all, the absorption of NOACs is variable. For example, the bioavailability of rivaroxaban is 60–80%, and that of apixaban is 50% higher than that of dabigatran. The latter, taken as a prodrug, has an oral bioavailability of just 6%, while the remainder passes through the intestine and, before being excreted in the feces, is two-thirds converted into an active drug by intestinal esterases. Despite the different levels of bioavailability, an important amount of active drug is excreted with the feces and, therefore, could have a local, as well as systemic, effect on a pre-existing mucosal vulnerability [11,31,32,33]. Another factor that must be taken into account when considering the pathophysiology of gastrointestinal bleeding in these anticoagulated patients is the knowledge that NOACs are substrates of a key efflux pump present along the gut, namely, the permeability glycoprotein (P-gp) [34,35]. This transporter, also called the ATP-binding cassette (ABC) subfamily member 1 (ABCB1) or multidrug resistance protein 1 (MDR1), is likely the actor of a protective mechanism against xenobiotics. It is present in different organs (e.g., kidney, liver, and blood-brain barrier) and, particularly, it is widely expressed along the intestinal epithelium, where it pumps such substances (e.g., drugs and toxins) back into the lumen [36,37]. Indeed, by transporting a series of substrates across intra- and extra-cellular membranes, P-gp is often involved in drug–drug interactions, as some drugs can either act as a substrate, an inducer, or an inhibitor of this glycoprotein. If NOACs are administered together with a P-gp inhibitor, the decrease in luminal efflux of the drug by this transporter will translate into a higher blood concentration of anticoagulant, thus increasing the bleeding risk [38]. Furthermore, P-gp is expressed in different concentrations along the bowel, with maximum expression in the distal ileum and minimum in the duodenal level. Consequently, the more distal segments appear to have a greater efflux of anticoagulant at the intraluminal level [39]. In this regard, some studies on surgical patients have been conducted on the effect of NOACs in patients undergoing bypass or major gastrointestinal resections, showing how these surgeries on the proximal gastrointestinal tract led to greater exposure to the anticoagulant in the more distal segments, which would then result in more sensitivity to NOAC topical damage [40]. Moreover, different in vitro studies [34,41] have been conducted so as to better characterize the expression of P-gp at the level of the intestinal epithelium.

### 2.2. The Role on Gastrointestinal Bleeding of ABC Transporters and Permeability Glycoprotein (P-gp)

Englund et al. [41] quantified the expression of a series of transporters of the ABC group along the gut using biopsies from volunteers undergoing routine examination and compared the results with in vitro analysis on an intestinal model derived from human colorectal adenocarcinoma cells. In this Swedish study [41], the authors found that P-gp levels were up to five-fold higher in the ileum compared to the duodenum or colon. Particularly, this intestinal model, which has been used in more than one study on NOAC gut-related pharmacokinetics, is composed of a particular cell line (Caco-2) [42,43] cultured under specific conditions so as to differentiate and (although being derived from the colon) resemble in phenotype the enterocytes of the small intestine to act as intestinal model, and expresses, among a number of transporters and enzymes, P-gp and breast cancer resistance protein (BCRP), which are both involved in NOAC pharmacokinetics. A comparative study by Gnoth et al. [44] on transport characteristics of rivaroxaban, both in vivo, using P-gp double-knockout mice vs. wild-type, and in vitro, studying the anticoagulant bidirectional efflux through Caco-2 model, wild-type and P-gp overexpressing cells, stresses that this NOAC is a substrate, but not an inhibitor, of P-gp, and that this mechanism can lead to drug–drug interactions (DDI). Hodin et al. [34] conducted a similar study using the same Caco-2 intestinal model to investigate in the case of all the NOACs’ transport via P-gp and BCRP. Three different ABC carrier-mediated transport profiles are identified: predominantly P-gp-dependent transport for dabigatran, preferential BCRP-dependent transport for apixaban, and approximately equivalent P-gp and BCRP-mediated transport regarding edoxaban and rivaroxaban. Zhang et al. [45], working on animal models, administered active charcoal to bile duct-cannulated dogs and rats receiving an intravenous dose of radiolabeled apixaban and studied drug transporter knockout rats and highlighted that, not only was apixaban undergoing intestinal excretion, but there was also a mechanism of entero–enteric recirculation of the anticoagulant via a mechanism of luminal excretion through efflux pumps followed by reabsorption. This finding gives further strength to the direct, topical damage of NOACs within the gut that has been described in literature. As mentioned before, a potentiation of systemic and topical damage can occur when NOACs are co-administered with drugs that are known substrates, inhibitors, or inducers of P-gp, BCRP, or cytochrome CYP3A4 (Figure 1).

## 3. Clinical Implications and Pharmacology

### 3.1. NOACs and Pharmacological Interactions

A large study by Kawano et al. [46] used Japanese insurance data to consider the bleeding risk for the combination of NOACs and antihypertensive medications. Particularly, patients under those antihypertensive drugs not acting on P-gp had a reduced bleeding risk. On the other hand, telmisartan, a known inhibitor of P-gp, led to higher bleeding rates, as the apixaban or rivaroxaban concentrations were increased in this case. A recent retrospective analysis by Hanigan and colleagues [47] on apixaban and rivaroxaban-associated bleeding risk in patients under combined P-gp and CYP3A4 inhibitors described an increased bleeding risk for some antiarrhythmics, such as amiodarone, dronedarone, diltiazem, and verapamil. DDIs aggravating the bleeding risk have also been observed in oncologic patients for some tyrosine kinase inhibitor (TKI)-NOAC combinations. Zhao et al. [48], for example, estimated the combination safety of rivaroxaban and TKIs relative to human cytochrome P450 metabolism and efflux transporters, showing increased bleeding risk for imatinib. Polypharmacy with drug combinations for antiviral treatments can also significantly alter NOACs efficacy, as the anticoagulant exposure increases more than two-fold when P-gp inhibitors of various potencies act simultaneously, thus increasing the bleeding risk at a systemic level [49,50]. Another systematic review published recently [50] on real world risks of DDIs with NOACs underlines, on the contrary, the occurrence of thrombotic events with some antiepileptics that were inducers of CYP3A4 only (phenytoin) and CYP3A4, together with P-gp (phenobarbital), suggesting lower NOAC concentrations in these patients. Knowledge about the potential effect of induction of transporters or CYP isoenzymes is limited within the literature. These drugs can alter, and in particular, limit the absorption of NOAC and favor its elimination. This, on the one hand, reduces the blood concentration [50] but, on the other hand, favors a greater concentration at the luminal level, thus exposing the intestinal surface to a greater risk of bleeding via a topical mechanism. The most critical interactions involving all NOACs also occur at the level of intestinal absorption and are attributable to potent P-glycoprotein (P-gp) inhibitors such as antifungals, macrolides, and antiretroviral protease inhibitors. Cardiovascular drugs include verapamil and amiodarone. Pharmaco-metabolic inducers of P-gp, such as rifampicin and ipperic, should not be administered in combination with NOACs [34]. In this regard, it has been observed that the levels of all NOACs increase when co-administered with potent intestinal P-gp inhibitors, such as fluconazole and ketoconazole. The P-gp inhibitor amiodarone itself causes a 60% and 40% increase in systemic exposure of dabigatran and edoxaban, respectively [1]. No data are available on the change in plasma concentrations of apixaban and rivaroxaban in the presence of amiodarone. However, it is important to mention that subgroup analysis of the ARISTOTLE [28] and ROCKET AF [27] studies, conducted with apixaban and rivaroxaban, respectively, showed no effect of amiodarone on the efficacy and safety of apixaban. By contrast, amiodarone reduces the efficacy of rivaroxaban and increases the risk of bleeding for the major bleeding [51]. The relevance of the transporter interaction at the level of absorption is well evidenced by the increased plasma levels of dabigatran when verapamil is administered 1h before dabigatran or in co-administration [48]. In contrast, no significant interaction is observed when verapamil is administered 2 h after dabigatran intake (increase in Cmax by about 10% and increase in area under the curve by about 20%) [48]. This is explained by the complete absorption of dabigatran after 2 h by P-gp inhibitors. As for edoxaban, co-administration with amiodarone results in a 40% increase in plasma concentrations of the anticoagulant. In this regard, it is interesting to note that the analysis of a subgroup of the ENGAGE AF-TIMI 48 [26] study shows that, in patients who were taking amiodarone at recruitment, the treatment regimen with low doses of edoxaban significantly reduced ischaemic events compared to patients not treated with amiodarone. In contrast, at higher doses of edoxaban, amiodarone did not lead to a significant efficacy and safety effect [50]. Together with the clinical data, plasma levels of the amiodarone–edoxaban combination are reported, documenting, for both dosages, an increase of approximately 30% in plasma concentrations [50]. As discussed by the authors, an increase in plasma concentrations caused by amiodarone in low-dose patients occurs at the inflection point of the plasma concentration–efficacy curve, resulting in a significant reduction in ischaemic events. In contrast, the increase in edoxaban concentrations in patients receiving amiodarone occurs at the flat part of the dose–effectiveness curve, preventing further reductions in ischemic events from being observed. Plasma levels of all NOACs decrease in the presence of P-gp inducers such as rifampicin, with which co-administration should be avoided.

### 3.2. Pharmacokinetic Features of NOACs

NOACs are characterised by both different volumes of distribution and different binding to plasma proteins. The volume of distribution of apixaban is small, suggesting distribution mainly in the systemic circulation, with limited extravascular localisation. In contrast, dabigatran is characterised by high hydrophilicity, poor plasma protein binding, and essential renal clearance, features that make this NOAC the only haemo-dialisable one. In this regard, it should be emphasised that dialysis is effective if performed within the first few hours of administration, otherwise the drug’s volume of distribution (60 L) prevents dialysis clearance of approximately 12 L when distribution is complete. An important pharmacokinetic feature distinguishing NOACs is the route of elimination, which is essentially renal for dabigatran, while it is both hepatic and renal for rivaroxaban, apixaban, and edoxaban. This leads to variations in the dosage and choice of NOACs based on the patient’s pathophysiological and demographic characteristics. For example, dabigatran is contraindicated in patients with a creatinine clearance <30 mL/min. Rivaroxaban and apixaban can be used with caution, always reducing the dose and monitoring the patient’s renal function. In patients with moderate renal impairment (creatinine clearance between 30 and 50 mL/min), dabigatran and rivaroxaban can be used at reduced doses, whereas apixaban, which is less renally eliminated, can be used at normal doses, at least in patients aged ≤80 years and weighing >60 kg.

All NOACs are, however, contraindicated in patients with severe hepatic impairment, while dose reduction is recommended for apixaban, rivaroxaban, and edoxaban when co-administered with potent CYP3A4 inhibitors, unlike dabigatran, which is not metabolised by cytochromes. Among the characteristics of NOACs, the elimination half-life of approximately 12 h to 14 h deserves consideration, a characteristic that suggests a dosage with double daily administration. The ratio between the maximum and minimum steady-state concentration in single daily administration is 4.5 for dabigatran, 10 for rivaroxaban, 10 for apixaban and 10–30 for edoxaban. The higher the ratio, the greater the fluctuation in plasma levels over 24 h.

The clinical consequences of these fluctuations can obviously lead to bleeding at the peak or thromboembolic events at the lowest concentrations. It is therefore plausible to try to minimise these variations by opting for twice a day administration and extended/controlled release forms. This option is also justified by the fact that a bid administration should benefit from a more constant maintenance of the plasma concentration of the NOACs and their actions, even in the event of variable drug exposure due to suboptimal treatment adherence. However, for rivaroxaban, it was preferred to opt for single administration. The high C-max levels with rivaroxaban, together with kinetics that are non-linear at doses >10 mg, which require intake with food, result in significant variability in peak concentrations. In this regard, a cross-over study was conducted comparing the pharmacokinetic and pharmacodynamic profile of apixaban 2.5 mg bid and rivaroxaban 10 mg/day 30 in the same patients. A mean C-max variability of 46% and 23% can be observed with rivaroxaban and apixaban, respectively. This extreme variability of rivaroxaban could be associated with the increased risk of gastrointestinal bleeding, compared to warfarin, observed in the ROCKET AF [27] clinical trial. To summarize, rivaroxaban [52] is mainly absorbed in the proximal small intestine, with dose-dependent bioavailability, and it is not linear with the dose administered. At the 10 mg dose, the estimated bioavailability is 80–100%, compared to 66% at the 20 mg dose, when administered on an empty stomach. The presence of food, probably by increasing its solubilisation and dissolution, significantly increases the bioavailability of rivaroxaban 20 mg, while also reducing the inter-individual variability of its plasma concentrations. It is important to remember that food intake implies a meal of at least 1300 Cal with 30–40% fat content. This is why it is essential to take rivaroxaban after meals. Apixaban [53] is predominantly absorbed in the distal part of the small intestine and in the ascending colon, reaching its peak concentration (C-max) 2–3 h after oral intake. Its bioavailability is approximately 50%, and approximately 35% of the unabsorbed portion is excreted with feces. Unlike rivaroxaban, intestinal absorption of apixaban is not affected by the presence of food. Similar considerations can be made regarding the absorption of edoxaban, which reaches C-max after 2 h and is not affected by the presence of food.

### 3.3. Pharmacodynamics of NOACs: Efficacy and Safety Considerations

From a pharmacodynamic point of view, NOACs have a direct effect that is maximal 2–3 h after their administration in accordance with the time to peak concentration and the direct correlation between plasma concentrations and anticoagulant effect. These characteristics distinguish them from warfarin, which, by inhibiting the activation of several coagulation factors, takes 3–5 days to manifest its anticoagulant action. This difference means that the initiation of NOAC treatment does not require a period of pre-treatment with heparin (‘bridging’). The same reasoning applies to the reversibility of the effect, which is much faster for NOACs, both because of their short half-life and the reversibility of their mechanism of action, than for warfarin. A detailed analysis of the bleedings observed in the different districts shows important differences between the different NOACs compared to warfarin. Dabigatran 150 mg bid (but not 110 mg bid) had a similar incidence to warfarin in cases of major bleeding (RE-LY) [25]. In the ROCKET AF [27] study, a comparable incidence of major bleeding was observed between rivaroxaban and warfarin. In contrast, the risk of bleeding was significantly lower for both apixaban (ARISTOTLE) [28] and edoxaban (ENGAGE) [26] compared to warfarin. Importantly, all NOACs demonstrated superiority over warfarin in intracranial haemorrhage. With regard to gastrointestinal bleeding, differences were observed between the various NOACs compared to warfarin. The administration of dabigatran 150 mg bid is associated with a higher and more significant incidence of gastrointestinal bleeding than warfarin. In particular, bleeding in patients treated with dabigatran was mostly referred to the lower part of the small intestine (53%) compared to those observed with warfarin (25%), in agreement with the activation of dabigatran from prophylactic to drug that occurs during the gastrointestinal route. Rivaroxaban causes a significantly higher increase in gastrointestinal bleeding than warfarin, probably attributable to its single-dose dosage with very high peak concentrations and extreme variability. In accordance with its site of absorption, bleeding occurs mainly in the proximal part of the small intestine. In contrast to dabigatran and rivaroxaban, apixaban is not significantly different in the risk of gastrointestinal bleeding compared to warfarin in terms of both incidence and site of bleeding. As for edoxaban, results from the ENGAGE AF-TIMI 48 [26] study show that the risk of gastrointestinal bleeding is significantly higher at high doses (60 mg/day) and lower at low doses (30 mg/day). In accordance with its kinetic profile, no differences are observed compared to warfarin in gastrointestinal bleeding sites. In clinical practice, the results of a recent Food and Drug Administration analysis report confirm an increased risk of gastrointestinal bleeding for rivaroxaban and dabigatran, in agreement with clinical studies. The authors of this pharmacovigilance study conclude that apixaban, among the NOACs, appears to be the drug with the best safety profile in terms of both major bleeding and gastrointestinal bleeding with equal efficacy. It is important to note that the pharmacovigilance data confirm those reported in the registration studies.

### 3.4. Focus on Dabigatran

Dabigatran capsules are made up of tartaric acid coated with dabigatran etexilate (a prodrug of dabigatran). Dabigatran differs from other NOACs in that it has a low bioavailability (estimated 6.5%), which results in significant variability in the amount absorbed [9]. In order to achieve oral absorption, dabigatran is administered as a prodrug (dabigatran etexilate), which, once it reaches the systemic circulation, is hydrolysed by hepatic and serum esterases and activated to dabigatran. Its absorption increases in an acidic environment, which is why the drug is formulated in the presence of tartaric acid. Studies at increasing doses of dabigatran show that the low oral bioavailability is not caused by a saturable first-pass process, as plasma concentrations increase linearly according to first-order, dose-independent kinetics. The capsules are designed for release in the stomach, and the molecule is absorbed in the distal small intestine. The unabsorbed drug passes through the gastrointestinal tract where it is mostly converted to dabigatran and eliminated with the feces. Many studies have reported that tartaric acid plays an important role in the induction of gastrointestinal bleeding. It has been suggested that tartaric acid adheres to the esophagus and damages the esophageal mucosa. However, the precise reason for gastrointestinal bleeding risk remains unknown. A recent study published by Kurokawa et al. [54] showed that dabigatran cytotoxicity can be prevented via antioxidant treatment. As expected, co-administration with H2 antagonists and proton pump inhibitors, which increase intestinal pH, leads to a lower solubilisation of dabigatran and a decrease in its absorption by 12% and 30%, respectively, but without changing its clinical efficacy. Administration with food significantly delays the drug’s absorption time, but without significantly affecting its bioavailability. It is therefore recommended that the drug should always be taken in the presence or absence of food [35].

## 4. Conclusions

Gastrointestinal bleeding is a frequent adverse event in patients treated with NOACs even if many studies underline that NOACs have a comparable risk to warfarin to develop gastrointestinal bleeding. Furthermore, patients suffering of gastroduodenal ulcers, diverticular disease, angiodysplasia, and hemorrhoids need a more careful medical prescription of NOACs. Apixaban resulted in a lower rate of gastrointestinal bleeding compared to dabigatran and rivaroxaban [55]. Studies’ data reported that gastrointestinal bleeding in patients treated with NOACs was less severe. More studies are needed to investigate this field and expand indications of NOACs in patients with thromboembolic cardiovascular conditions.

## Data Availability

Not applicable.
